# Effects of the Connections program on return‐to‐custody, mortality and treatment uptake among people with a history of opioid use: Retrospective cohort study in an Australian prison system

**DOI:** 10.1111/add.16339

**Published:** 2023-09-19

**Authors:** Elizabeth Sullivan, Reem Zeki, Stephen Ward, Juanita Sherwood, Marc Remond, Sungwon Chang, Kypros Kypri, James Brown

**Affiliations:** ^1^ College of Health, Medicine and Wellbeing University of Newcastle Callaghan NSW Australia; ^2^ Justice Health and Forensic Mental Health Network Malabar NSW Australia; ^3^ University of Technology Sydney Ultimo NSW Australia

**Keywords:** Australia, cohort study, evaluation, linkage, mortality, opioid agonist treatment, opioids, prison, recidivism, throughcare

## Abstract

**Background and Aims:**

Connections is a voluntary health program that facilitates access to opioid agonist treatment (OAT) and social services for people with opioid use exiting prison. This study aimed to measure the effectiveness of Connections in reducing recidivism and improving health outcomes for people with a history of opioid use on leaving prison.

**Design:**

Retrospective cohort study with quasi‐random allocation to the program.

**Setting:**

Public adult prisons in New South Wales, Australia, 2008–2015.

**Participants:**

Adults released from custody with a history of opioid use. Of 5549 eligible releasees, 3973 were allocated to Connections and 1576 to treatment‐as‐usual.

**Measurements:**

Outcomes were return‐to‐custody, all‐cause mortality, and OAT participation.

**Findings:**

Regression analyses on an intention‐to‐treat basis, and adjusting for baseline propensity scores, comparing patients allocated to Connections versus treatment‐as‐usual showed no difference in rates of return‐to‐custody within 2 years (hazard ratio [HR]: 1.01; 95% confidence interval [CI]: 0.92 –1.12). Patients allocated to the Connections program were more likely to access OAT (odds ratio [OR]: 1.21; 95% CI: 1.06–1.39) and had lower mortality within 28 days of release (0.25% vs. 0.66%; HR: 0.38; 95% CI: 0.14–1.03). Differences in mortality did not persist beyond 28 days. Subgroup analyses showed that allocation to Connections was associated with higher risk of return‐to‐custody within 28 days for Aboriginal and/or Torres Strait Islander (Indigenous) and female releasees.

**Conclusions:**

The Connections program for people with opioid use exiting prison did not reduce the likelihood of return‐to‐custody but did facilitate opioid agonist treatment participation on release from prison.

## INTRODUCTION

In 2020, there were 41 060 adults incarcerated in Australian prisons, one in seven of whom were sentenced for an illicit drug offence [1]. Australia’s incarceration rate of 202 per 100 000 population [1] is one third of that in the United States (US) (639 per 100 000) [2] but higher than rates in England and Wales (173 per 100 000) [3] and Canada (104 per 100 000) [4, 5].

People in prison suffer more infectious and chronic disease than the general population, typically have histories of trauma [6], and have a high prevalence of mental illness [7, 8, 9]. Australian survey data show that approximately half of people in prison had received psychiatric care before entering custody [9]. On leaving prison, many releasees struggle to reconnect with family, access healthcare, secure employment and find suitable accommodation [10]. This is at least partly because of system‐level barriers including being prohibited from some jobs and housing because of their criminal record. Poor mental health, often because of drug use, often compounds their struggles [6, 11, 12]. The nexus of poverty, social isolation and addiction explains the high mortality on leaving prison [13, 14, 15, 16], from homicide, suicide and unintentional drug overdose [6, 13, 14, 15, 16].

Criminal activity associated with drug use after release from prison increases recidivism, leading to the so‐called ‘revolving door at the prison gate’ phenomenon [17]. In 2009, the New South Wales (NSW) Bureau of Crime Statistics and Research (BOCSAR) estimated that a 10% reduction in the imprisonment rate would save the state $30 million annually [18]. Nonetheless, the prison population increased by a third in the following decade [19, 20].

NSW is Australia’s most populous state (8.2 million people in 2021). It encompasses an area of 802 000 km^2^, making it more than twice the size of Germany and 15% larger than the US state of Texas. The state prison system comprises 37 public prisons [21] holding 11 029 inmates in September 2021 [22], and one private prison that holds ~10% of the prison population [21, 23]. NSW’s imprisonment rate of 197 per 100 000 adults is similar to that of Australia as a whole [24].

In 1999, the NSW Drug Summit recommended that there be greater cooperation between government and non‐government organizations to provide pre‐release and post‐release continuity of care, treatment and rehabilitation for people with a history of drug problems [25]. Summit findings of high rates of drug overdose and death in people released from prison prompted the establishment of a pilot program in 2007 that was formalized as Connections in 2011. Connections is run by the Justice Health and Forensic Mental Health Network (Justice Health NSW), a statewide network that delivers healthcare across custodial, forensic mental health, hospital and community settings [26].

Connections targets people in prison who have a history of drug problems, providing pre‐to‐post‐release throughcare. Clinical Support Workers are assigned to each participant to facilitate engagement with health services and community follow‐up in the 4 weeks after release from prison [27]. Participants who complete Connections receive a pre‐release assessment and treatment plan, at least one face‐to‐face or telephone contact post‐release and a follow‐up assessment 4 weeks after release. In a preliminary analysis, we found that releasees who completed Connections post‐release were substantially less likely to return‐to‐custody within 2 years than releasees who had only participated pre‐release [24, 28]. That finding gave cause for optimism about the effectiveness of Connections, but required further investigation to account for possible selection bias. For example, it is likely that releasees who completed Connections differed at baseline from those who did not complete it in ways that may at least partly explain their lower rate of recidivism. In addition, the effects of the program on health outcomes were unknown.

Accordingly, our aim was to evaluate the effectiveness of Connections in reducing recidivism and improving health outcomes for people with a history of opioid use on leaving prison [29]. We aimed to estimate the effect of the intervention regardless of the level of engagement with the program. We took this approach to reflect the real‐world context, in which participation in Connections is voluntary and the level of engagement depends on numerous factors including individual motivation. We address the research questions:

1. Is allocation to Connections associated with a lower rate of return‐to‐custody within 2 years of release?
2. Is allocation to Connections associated with a lower rate of mortality within 2 years of release?
3. Is allocation to Connections associated with greater opioid agonist treatment (OAT) participation (commencement of, or retention in, OAT) in the 2 years after release?
4. Do outcomes associated with allocation to Connections differ between men and women?
5. Do outcomes associated with allocation to Connections differ between Aboriginal and/or Torres Strait Islander (hereafter referred to as Indigenous) versus non‐Indigenous participants?

## METHODS

### Setting

We conducted the study using data collected in all NSW public prisons from 2008 to 2015.

### Design

In a published protocol, we pre‐specified the design of a population‐based, retrospective cohort study evaluating the effects of Connections [29]. Although people were not randomly allocated to Connections or treatment‐as‐usual, they were allocated in a way we judged unlikely to produce systematic differences between the groups at baseline (e.g. based on severity of their opiate use or length of sentence). Rather, State budget allocations dictated when Connections was available such that allocation was determined solely by the availability of places on the program rather than characteristics of eligible individuals due for release. It is, therefore, reasonable to expect little selection bias, a common source of error in non‐randomized studies [30]. We nonetheless adopted a propensity score (PS) matching approach (described below) to mitigate any differences in baseline characteristics between the intervention and control groups on variables associated with the outcomes of interest and to enable adjustment for any differences.

**FIGURE 1:**
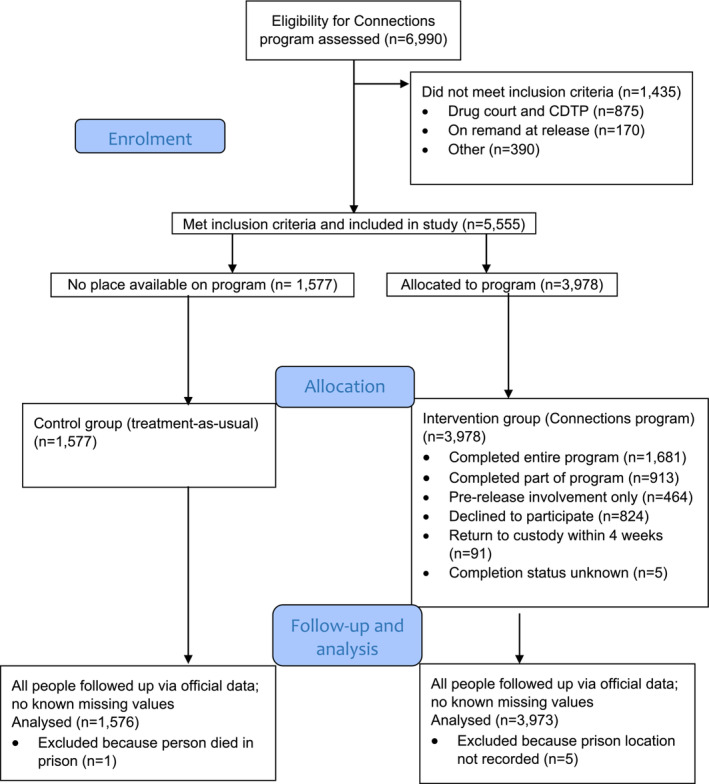
Flow of people through the Connections program evaluation.

Given the aim to estimate the effects of a novel program compared to treatment‐as‐usual, and the quasi‐random allocation to exposure, the study has many characteristics of a trial [24]. We, therefore, adapted the standard Consolidated Standards of Reporting Trials diagram to represent the passage of people through the study according to whether they were allocated to Connections or treatment‐as‐usual (Figure 1). Note that, as the primary research question and analysis plan were not preregistered on a publicly available platform, results should be considered exploratory.

### Eligibility and allocation to Connections

In accordance with the study protocol [29], people were eligible for Connections if they: (1) were receiving OAT in custody; or (2) had ceased having OAT in the 6 months before release; or (3) were pregnant or gave birth during the current period of incarceration (or in the 6 months before entering custody) and had a history of drug problems; or (4) were not on OAT, but had engaged with Drug and Alcohol Services for treatment.

During periods when staffing and other resources permitted Connections to accept new participants, eligible releasees were allocated to the program whereupon they could choose whether to participate. Eligible people received treatment‐as‐usual rather than Connections during periods when: (1) the service lacked the capacity to enrol patients (55%); (2) referrals were late or release from custody occurred earlier than expected (35%); and for other reasons relating to service delivery capacity (10%) [29]. Unpublished Corrective Services data show dramatic increases in referrals at various points, which rapidly overran capacity (e.g. in 2011, there were not places on the Connections program for 30% of the patients eligible to be referred).

### Intervention

Connections provides practical support for people with a history of drug problems. It is a voluntary throughcare program [29] focused on the transition from prison and engages with people in the lead‐up to release and in the 28 days post‐release. Connections uses a strengths‐based approach with a trained Clinical Support Worker allocated to each participant as a care manager. The aim is to develop support for the participant, identify their strengths and to assertively make links with health and social services to facilitate the releasee’s re‐entry into the community, thereby decreasing their risk of coming to harm or reoffending in the vulnerable period immediately after release [31, 32]. Once a releasee agrees to participate, a Clinical Support Worker assesses them face‐to‐face or by telehealth. Self‐determination is a key element of the strengths‐based approach [31], so participants were encouraged to identify their needs and goals for the post‐release period and to develop a release plan. This may have included facilitating access to OAT and other health services, linking participants with social services, giving them access to educational opportunities, helping them obtain identification documents, providing food and clothing and assistance in finding housing [32].

Connections was designed to be flexible, so the amount and type of support offered varied according to participant needs. For example, on release, a participant may have been picked up by a Clinical Support Worker and driven to a welfare services office to obtain identification documents and a Medicare card, and then to their OAT appointment. Other participants with existing support networks may have only needed a phone call from a support worker to check if they had accessed services.

Clinical Support Workers were located across NSW and Connections was available in all public and private adult prisons, subject to annual state budget allocations. Clinical Support Workers typically had professional qualifications in health, psychology or social work and received orientation, mentoring and clinical supervision. Training of the team in health, welfare and cultural engagement occurred over 2 days bi‐annually. Resources required for the program included access to telephones, motor vehicles, up‐to‐date local area treatment plans outlining services available in each geographical area and standardized pre‐ and post‐program assessment procedures and materials.

### Control condition: Treatment‐as‐usual

People who were not allocated to Connections received no contact from Justice Health NSW staff on release from prison.

### Data

The Centre for Health Record Linkage (CHeReL) linked the records of eligible people to 10 health, mortality and justice datasets (for details see the published protocol) [29]. In this study, we used four datasets, all of which provided at least 2 years of follow‐up data from the last date on which participants were enrolled in Connections (i.e. up to 31 December 2017):

1. ‘Connections dataset’, which contained records of eligible people due for release from 1 January 2008 to 31 December 2015. Variables include age, sex, Indigenous status, whether invited to join the program and level of engagement with the program.
2. BOCSAR custody dataset including reception date, release date, previous and subsequent episodes of incarceration.
3. Registry of Birth Deaths and Marriages, which records deaths occurring in NSW. If a person whose ‘usual residence’ was in NSW died in another Australian state, their death was included in this dataset. People who died in other countries, or in other Australian states with usual residence outside NSW, were not included in this dataset.
4. Electronic Recording and Reporting of Controlled Drugs System‐Methadone Subsystem dataset, which provided information on each individual’s engagement with OAT in prison and the community.

### Outcomes

**FIGURE 2:**
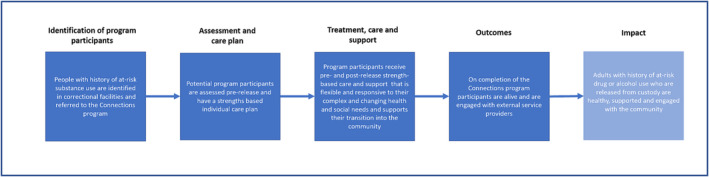
Diagrammatic representation of the components and objectives of the Connections program.

In the study protocol, we specified return‐to‐custody as the primary outcome in keeping with the NSW Government’s rationale for funding of the Connections program as a means of reducing recidivism [27, 29]. However, our in‐depth analysis of the program (see Figure 2) suggested that it was, in practice, more focused on keeping people alive during the weeks after release from prison, when mortality is high. This was to be achieved via greater engagement with health and social services and OAT. Before analysing data, we considered all‐cause mortality and OAT participation to be more suitable outcomes for evaluating the program. However, to avoid any appearance of post hoc selectivity, we adhere to the pre‐specified protocol analyzing return‐to‐custody as the primary outcome, and analyse all‐cause mortality and OAT participation as secondary outcomes. In this paper, we do not examine the other secondary outcomes described in the protocol (i.e. all‐cause hospitalisation, episodes of mental health problems and drug and alcohol or emergency care) because of lack of space.

Conservatively, we present the results as effect estimates with confidence intervals rather than applying significance tests. The results, therefore, indicate the size of the estimated intervention effect and the degree of precision in that estimate, rather than a simplistic rejection or failure to reject null hypotheses [28, 33, 34].

### Analysis

We classified eligible people who were offered a place on the Connections program as the intervention group (‘Connections’) and eligible people who were not offered a place on the program as the control group (‘treatment‐as‐usual’). Given that some people left prison more than once during the study period, we used each person’s last eligible episode of imprisonment in the analysis, so that no individual is represented more than once in the analyses.

We applied an intention‐to‐treat approach comparing the two groups on the basis of allocation (see Figure 1) regardless of whether people in the intervention group engaged with or completed the Connections program [30]. Therefore, the intervention group comprised all individuals allocated to Connections whatever their subsequent level of participation in the program. It includes those who were allocated to the program, but chose not to participate. We also estimated effects of the intervention in important subgroups, namely, women and Indigenous people, as we pre‐specified in the study protocol [29].

In addition to comparing groups according to quasi‐random allocation to exposure, we sought to minimise bias from residual differences between groups at baseline through propensity scoring, weighting by odds in the final model [35], giving people allocated to Connections a weight of 1 and people allocated to treatment‐as‐usual a weight equal to their PS odds, that is, PS/(1‐PS) [35]. We included as covariates in the PS model variables in the linked datasets that, if they influenced Clinical Support Worker decisions in offering places on the Connections program, could bias effect estimates. Having no a priori basis for model specification, we used decision trees to identify candidate variables for the PS model, and then forward selection stepwise logistic regression (inclusion *P*‐value of 0.10) to estimate associations according to group allocation. Variables we tested were discharge year, releasee gender, Indigenous status, age category, whether the releasee received OAT in prison, prison remoteness, number of times in custody, length of current imprisonment in months and Level of Service Inventory‐Revised (LSI‐R) category, a measure of recidivism risk [36]. Table S1 summarises the PS matching variables (with *n* and percentages for raw data and the weighted sample), demonstrating that the Connections and treatment‐as‐usual groups are well‐represented in all categories or levels of the variables. Standardised differences confirm the effectiveness of weighting to correct differences in covariate distributions between the groups. Table S2 summarises PS distributions in the study groups and the weights applied to the treatment‐as‐usual group, showing overlap in the scores and a weight distribution that, relative to the mean, did not require adjustment.

We pre‐specified binary logistic regression to address research questions 1 and 2 [29], but later realised that we should analyse outcomes in the specified post‐release periods, namely, within 28, 29 to 91, 92 to 183 days and 2 years of release. Accordingly, we used Cox proportional hazards regression, which accounts for the time dependence, estimating crude and weighted hazard ratios (HRs) and 95% confidence intervals (CIs) for return‐to‐custody and mortality.

The start of the follow‐up period for Cox regression models was date of release. For return‐to‐custody, the end date was date of return‐to‐custody within the specified post‐release period (e.g. 28 days) if an individual returned to custody. Otherwise, the censoring date was date of death for individuals who died within the specified post‐release period, or the date marking the end of the post‐release period or the end of the data series (31 March 2018) for individuals who did not return‐to‐custody or die within the specified period. For individuals who, after their final release, did not return‐to‐custody or die before the end of the data series, we verified the data against BOCSAR court appearances records. For individuals with ‘imprisonment’ recorded as type of penalty, we used the date of the most serious offence to define return‐to‐custody. For individuals who died, the end date was date of death within specified post‐release periods. Otherwise, the censoring date was the end of the specified post‐release period or 31 March 2018 for individuals who had not died. We undertook survival analysis using Kaplan‐Meier techniques to examine the effect of the intervention on return‐to‐custody and mortality within 2 years.

To address research question 3, we used complex sample logistic regression with PS weighting to estimate effects of the intervention on OAT attendance within 28 days of release. These produced estimates of crude and weighted odds ratios (ORs) with 95% CIs.

For questions 4 and 5, we used weighted Cox proportional hazards regression to estimate crude and weighted HRs and 95% CIs for return‐to‐custody, and mortality, within 28 days and 2 years, by group, stratified by Indigenous status and then sex. We used logistic regression to estimate effects of the intervention on OAT attendance within 28 days of release, estimating crude and weighted ORs with 95% CIs for Indigenous and non‐Indigenous participants, and for women and men. We used PS weighting in stratified analyses, excluding the stratifying variables from the model relating to that variable (e.g. excluding sex from the PS model stratifying by sex).

## RESULTS

From 2008 to 2015, 5555 releasees were deemed eligible to participate in Connections, of whom 5549 were included in the final analysis. Of the six people excluded, one had died in prison and was, therefore, unable to participate, and records for the other five did not specify which prison they were released from, which was necessary for the PS model.

### Demographics and program participation

Table 1 summarises demographic characteristics, prison history and receipt of OAT in prison, among the 5549 releasees allocated to Connections or treatment‐as‐usual: 940 (16.9%) were female, 1538 (27.7%) were Indigenous and 3973 (71.6%) were allocated to Connections. Of those allocated to Connections, 67.5% completed the program, 23.0% partially completed it, 20.7% declined to participate, 11.7% were lost to follow‐up and 2.3% returned to custody within 28 days of release.

**TABLE 1:** Characteristics of people allocated to the Connections program and to treatment‐as‐usual.

Characteristic	Allocated to Connections program, (*n* = 3973)	Allocated to treatment‐as‐usual, (*n* = 1576)
	*n* (%)	*n* (%)
Age, years		
<25	323 (8.1)	139 (8.8)
25–39	2417 (61)	973 (62)
>39	1233 (31)	464 (29)
Sex		
Female	701 (18)	239 (15)
Male	3272 (82)	1337 (85)
Indigenous status		
Indigenous	1087 (27)	451 (29)
Not Indigenous	2886 (73)	1125 (71)
No. of previous periods in prison		
0	470 (12)	170 (11)
1–4 times	1856 (47)	765 (49)
5 or more times	1647 (42)	641 (41)
Received OAT in prison		
Yes	2833 (71)	1055 (67)
No	1140 (29)	521 (33)

Abbreviation: OAT, opioid agonist treatment.

### Analysis of primary and secondary outcomes

**FIGURE 3:**
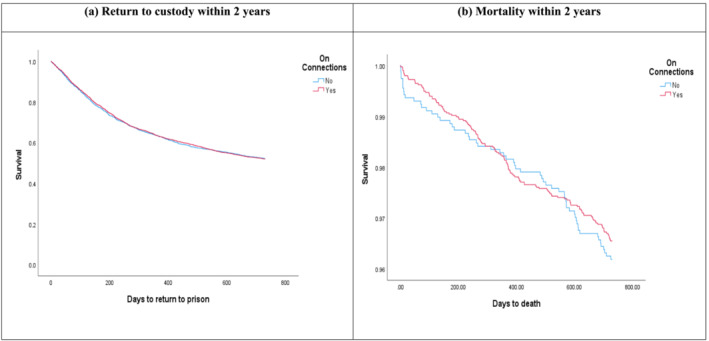
Survival curve for (a) return‐to‐custody and (b) mortality, within 2 years of release.

Table 2 presents numbers of people who returned to custody or died within 2 years of release from prison. It also shows HRs reflecting the weighted effect of the Connections program on these outcomes. Proportions of each group who returned to custody were similar at each time point and weighted proportions and HRs do not suggest any effect of the intervention on return‐to‐custody rates (within 28 days of release HR = 0.99; 95% CI = 0.71–1.39; within 2 years of release HR = 1.01; 95% CI = 0.92–1.12) (see also Figure 3a). The proportion that died within 28 days of release was considerably lower in those allocated to Connections (0.25%) than treatment‐as‐usual (0.66%). However, the CI for the point estimate is wide (HR = 0.38; 95% CI = 0.14–1.03), reflecting relatively small numbers of deaths, and any effect did not persist beyond 28 days post‐release (within 2 years HR = 0.99; 95% CI = 0.68–1.44) (see also Figure 3b).

**TABLE 2:** Return‐to‐custody and all‐cause mortality within 2 years of release from prison.

	N	Unweighted %	Weighted %	HR (95% CI)^a^
(a) Returned to custody …				
Within 28 days of release				
On Connections (*n* = 3973)	162	4.1	4.1	0.99 (0.71–1.39)
Treatment‐as‐usual (*n* = 1575)	69	4.4	4.6	1
29–91 days after release				
On Connections (*n* = 3801)	353	9.3	9.3	0.96 (0.77–1.19)
Treatment‐as‐usual (*n* = 1496)	140	9.4	9.7	
92–183 days after release				
On Connections (*n* = 3438)	413	12.0	12.0	0.94 (0.77–1.16)
Treatment‐as‐usual (*n* = 1353)	173	12.8	12.6	
Within 2 years of release				
On Connections (*n* = 3973)	1889	47.5	47.5	1.01 (0.92–1.12)
Treatment‐as‐usual (*n* = 1575)	744	47.2	47.0	1
(b) Died …				
Within 28 days of release				
On Connections (*n* = 3973)	10	0.25	0.25	0.38 (0.14–1.03)
Treatment‐as‐usual (*n* = 1575)	10	0.63	0.66	1
29–91 days after release				
On Connections (*n* = 3963)	11	0.28	0.28	0.83 (0.21–3.23)
Treatment‐as‐usual (*n* = 1565)	4	0.26	0.33	1
92–183 days after release				
On Connections (*n* = 3952)	18	0.46	0.46	1.78 (0.52–6.11)
Treatment‐as‐usual (*n* = 1561)	5	0.32	0.26	1
Within 2 years of release				
On Connections (*n* = 3973)	137	3.45	3.44	0.99 (0.68–1.44)
Treatment‐as‐usual (*n* = 1575)	60	3.81	3.48	1

Abbreviations: CI, confidence interval; HR, hazard ratio.

^a^ Hazard Ratio from the complex sample Cox regression using the propensity score weighting. We present variables included in the model in Table S1.

Table 3 presents the numbers and proportions of people who presented for OAT in the 28 days after release from prison. People allocated to Connections were more likely to present for OAT than people receiving treatment‐as‐usual (OR = 1.21; 95% CI = 1.06–1.39).

**TABLE 3:** Started or continued opioid agonist treatment in the community within 28 days of release from prison.

	No.	Unweighted %	Weighted %	OR^a^ (95% CI)
On Connections (*n* = 3973)	2454	61.8	61.8	1.21 (1.06–1.39)
Treatment‐as‐usual (*n* = 1575)	851	54.0	57.1	1

Abbreviations: CI, confidence interval; OR, odds ratio.

^a^ Calculated from the complex sample logistic regression using the propensity score weighting. We present variables included in the model in Tables S1 and S2.

### Subgroup analyses

Pre‐specified subgroup analyses suggest that for Indigenous releasees (results presented in Table S3), allocation to Connections increased the risk of return‐to‐custody within 28 days (HR = 2.30; 95% CI = 1.14–4.65), but that increased risk did not persist at 2 years (HR = 1.14; 95% CI = 0.97–1.35). We did not find greater OAT utilisation within 28 days of release in Indigenous people allocated to Connections (OR = 1.10; 95% CI = 0.85–1.41) (Table S4).

Women allocated to Connections were at much greater risk of returning to prison within 28 days of release (HR = 10.9; 95% CI = 2.57–46.1) (Table S5), however, this did not persist at the 2 year follow‐up (HR = 1.29; 95% CI = 0.95–1.76). We did not find evidence of intervention effects on mortality within 28 days (HR = 0.78; 95% CI = 0.07–8.68) or 2 years (HR = 1.05; 95% CI = 0.39–2.81) (Table S5), or in OAT utilisation among women (OR = 0.89; 95% CI = 0.63–1.27) (Table S6).

### Sensitivity Analysis

In a post hoc sensitivity analysis, we excluded 824 people allocated to Connections who declined to participate, finding the same pattern of results (Table S7).

## DISCUSSION

### Key findings

People leaving prison with a history of opiate use who were allocated to the 28‐day Connections program had greater uptake of OAT than people who received treatment‐as‐usual (i.e. no further contact). Although results suggested that people allocated to Connections had lower mortality within 28 days of release than those receiving treatment‐as‐usual (0.25% vs 0.66%), numbers were small and the difference did not persist. Allocation to the Connections program did not reduce the rate of return‐to‐custody.

### Mortality, OAT and return‐to‐custody

Our findings in relation to OAT uptake are consistent with a systematic review of opioid‐related treatment outcomes among incarcerated people in high income countries [37]. That review concluded that such treatment programs were associated with greater adherence to OAT, as observed in our study. However, in contrast to the review finding of lower re‐incarceration, mortality and non‐fatal overdose rates among program participants [37], we did not find that allocation to Connections was associated with a decline in return‐to‐custody, nor a persistent reduction in mortality. That review did not assess the risk of bias in the primary studies, most of which used standard cohort designs [37]. Allocating people to Connections or to treatment‐as‐usual based on the availability of places on the program, without reference to their individual characteristics (i.e. quasi‐randomly), minimised selection bias, permitting less biased effect estimates than are produced with standard cohort studies [24].

OAT has been shown to be effective in increasing abstinence, reducing mortality and improving the physical and mental health of people with opiate use [38, 39]. It is, therefore, encouraging to find that people allocated to Connections were more likely to engage with OAT within 28 days of release than those who received treatment‐as‐usual. This may partially explain the lower 28‐day mortality in the Connections group compared to treatment‐as‐usual and is consistent with findings of a large record‐linkage study in NSW that receiving OAT was associated with lower mortality in opiate users, particularly from drug overdose [40].

The period following cessation of OAT is associated with high mortality, particularly in the first 28 days [41, 42]. Our point estimate suggesting an initial protective effect of the Connections program against this outcome warrants further consideration. There may be value in extending the program beyond 28 days, however, the finding needs replicating given the uncertainty of our estimate. Allocation to Connections was not associated with a reduction in the rate of return‐to‐custody within 2 years of release, but it should be noted that desistance was not an explicit goal of Connections.

### Findings for women and Indigenous participants

Women comprised 17% of our study cohort compared to 7% of the NSW adult prison population [38]. This is consistent with a 2015 survey of the NSW prison population showing that among respondents who reported ‘ever using methadone or buprenorphine’, 47% of women reported daily use within the 12 months before incarceration compared to only 30% of men [9]. A higher proportion of women (16%) than men (10%) reported that they required assistance to quit opioids [9].

Our subgroup analyses suggest that participation in Connections did not increase post‐release OAT engagement among women and Indigenous people and may have increased the risk of return‐to‐custody. These poorer outcomes possibly reflect the fact that the program was not designed to be gender‐specific or culturally appropriate for Indigenous people. A recent investigation of Indigenous women’s experiences of healthcare in NSW prisons reported that institutional racism, discrimination, stereotyping and loss of autonomy were barriers to accessing healthcare [43]. The authors concluded that a decolonizing approach, including enhanced access to Indigenous community‐controlled health services in prison, is required to address this inequity [43].

Similarly, a recent review of post‐release programs for women with substance use problems reported that of 12 programs involving 3799 women, only five were associated with a significant reduction in recidivism, and only one was associated with a significant reduction in substance use [44]. The authors concluded that women in prison with substance use problems benefit most from continuity of care from prison to the community and that such care must incorporate gender‐responsive programming and individualised case management to target comorbid mental health and substance use problems [44].

The rate of incarceration of women in NSW has increased significantly in recent years [45] while Indigenous people continue to be over‐represented in the Australian criminal justice system [46]. Our subgroup analyses showing that Indigenous women allocated to Connections fared worse than Indigenous women receiving treatment‐as‐usual highlight the need to consider how the needs of this group can be better addressed.

### Limitations

Our choice of return‐to‐custody within 2 years as the primary outcome was driven by the NSW government’s original characterisation of the Connections program. It is perhaps unrealistic to expect effects up to 2 years later from a program that supports participants for up to 28 days after they are released from prison.

Connections operates by linking releasees to services also funded by state government. It is, therefore, plausible that the effectiveness of the system response is diminished when less funding is available. Under certain conditions, such a process would bias effect estimates in favour of Connections because people allocated to treatment‐as‐usual would tend to receive less input from community services than they would have received during periods in which people were being allocated to Connections. Any such effects depend on (1) the extent to which releasees access services in the absence of the Connections program; and (2) how much those services affect outcomes.

We considered the possibility that being on Connections would expose participants to greater surveillance by authorities, increasing the crime detection rate and thereby, the likelihood that participants would be returned to custody. We judged that scenario unlikely given that Justice Health’s Connections Case Support Workers have no role in law enforcement or surveillance.

We had no data regarding the exact nature and intensity of intervention, or participant adherence to the program. It was, therefore, impossible to determine to what extent effect estimates are related to how well Connections was implemented.

## CONCLUSION

Being allocated to Connections increased engagement with OAT in the 28 days after release from prison. The lack of clear impact on mortality and recidivism indicates a need for strategies to address substance use problems, but also housing, employment and social isolation for people leaving prison.

## AUTHOR CONTRIBUTIONS

**Elizabeth Sullivan:** Conceptualization (lead); formal analysis (equal); funding acquisition (lead); investigation (equal); methodology (equal); project administration (lead); resources (lead); supervision (lead); writing—original draft (lead); writing—review and editing (equal). **Reem Zeki:** Conceptualization (supporting); data curation (equal); formal analysis (equal); investigation (supporting); project administration (equal); writing—original draft (supporting); writing—review and editing (equal). **Stephen Ward:** Conceptualization (equal); funding acquisition (supporting); project administration (supporting); writing—original draft (equal); writing—review and editing (equal). **Juanita Sherwood:** Conceptualization (equal); formal analysis (supporting); writing—original draft (equal); writing—review and editing (equal). **Marc Remond:** Formal analysis (supporting); project administration (equal); writing—original draft (equal); writing—review and editing (equal). **Sungwon Chang:** Conceptualization (equal); formal analysis (supporting); funding acquisition (equal); writing—original draft (supporting); writing—review and editing (supporting). **Kypros Kypri:** Conceptualization (lead); investigation (equal); methodology (lead); writing—review and editing (lead). **James Brown:** Conceptualization (equal); data curation (equal); formal analysis (lead); investigation (equal); methodology (equal); software (equal); supervision (equal); writing—original draft (equal); writing—review and editing (equal).

## DECLARATION OF INTERESTS

The Connections program is run by the Justice Health and Forensic Mental Health Network (Justice Health NSW), of which E.S., R.Z. and S.W. are employees. No authors are involved in the day‐to‐day functioning of the Connections program.

## ETHICS STATEMENT

The Aboriginal Health and Medical Research Council of NSW (HREC/1187/16), NSW Population and Health Services Research Ethics Committee (HREC/16/CIPHS/17), Justice Health NSW Human Research Ethics Committee (HREC/16/JH/15), Corrective Services NSW Ethics Committee (D16/569544) and University of Technology Sydney Human Research Ethics Committee (ETH18–2587) approved the study protocol. The University of Newcastle Human Research Ethics Committee ratified those approvals (H‐2020‐0074).

## Supporting information


**Table S1.** Variables in the propensity score matching for people on Connections versus treatment‐as‐usual.
**Table S2.** Probabilities of allocation to Connections versus treatment‐as‐usual, and distribution of propensity score weights for releasees allocated to treatment‐as‐usual.
**Table S3.** Return‐to‐custody and all‐cause mortality within 28 days and within two years of release, by Indigenous status.
**Table S4.** Started or continued OAT in the community within 28 days of release from prison, by Indigenous status.
**Table S5.** Return‐to‐custody and all‐cause mortality within 28 days and within two years of release, by sex.
**Table S6.** Started or continued OAT in the community within 28 days of release from prison, by sex.
**Table S7.** Sensitivity analysis excluding people who were allocated to Connections but who declined to participate: Return‐to‐custody and all‐cause mortality within 2 years of release.

## Data Availability

Author elects to not share data as ethical approvals for this project require that the linked data used in this analysis not be shared to protect privacy and confidentiality.
