# Supplementary material for: Effects of the Connections program on return‐to‐custody, mortality and treatment uptake among people with a history of opioid use: Retrospective cohort study in an Australian prison system
Source: Addiction. 2023 Sep 19;119(1):169–79. doi: 10.1111/add.16339 (PMC10952213; doi:10.1111/add.16339)
Supplement: Supplementary file 1 — Table S1. Variables in the propensity score matching for people on Connections versus treatment‐as‐usual. Table S2. Probabilities of allocation to Connections versus treatment‐as‐usual, and distribution of propensity score weights for releasees allocated to treatment‐as‐usual. Table S3. Return‐to‐custody and all‐cause mortality within 28 days and within two years of release, by Indigenous status. Table S4. Started or continued OAT in the community within 28 days of release from prison, by Indigenous status. Table S5. Return‐to‐custody and all‐cause mortality within 28 days and within two years of release, by sex. Table S6. Started or continued OAT in the community within 28 days of release from prison, by sex. Table S7. Sensitivity analysis excluding people who were allocated to Connections but who declined to participate: Return‐to‐custody and all‐cause mortality within 2 years of release. [file ADD-119-169-s001.docx]

**Supplementary Table 1. Variables in the propensity score matching for people on Connections versus treatment-as-usual**

| **Variables in the propensity score model** | **Number** | | **Proportion (%)** | | | **Standardized difference** | |
| --- | --- | --- | --- | --- | --- | --- | --- |
|  | ***On Connections*** | ***Treatment-as-usual*** | ***On Connections*** | ***Treatment-as-usual (Unweighted)*** | ***Treatment-as-usual (Weighted)*** | ***Unweighted*** | ***Weighted*** |
| **Age group, years** |  |  |  |  |  |  |  |
| <25 | 323 | 139 | 8.1 | 8.8 | 8.7 | -2.5 | -2.2 |
| 25-29 | 722 | 280 | 18.2 | 17.8 | 17.4 | 1.0 | 2.1 |
| 30-34 | 860 | 374 | 21.6 | 23.7 | 20.9 | -5.0 | 1.7 |
| 35-39 | 835 | 319 | 21 | 20.2 | 21.2 | 2.0 | -0.5 |
| 40-44 | 621 | 250 | 15.6 | 15.9 | 15.6 | -0.8 | 0.0 |
| 45-49 | 388 | 137 | 9.8 | 8.7 | 10.7 | 3.8 | -3.0 |
| 50-54 | 149 | 61 | 3.8 | 3.9 | 3.4 | -0.5 | 2.1 |
| >54 | 75 | 16 | 1.9 | 1 | 2 | 7.5 | -0.7 |
|  |  |  |  |  |  |  |  |
| **Sex** |  |  |  |  |  |  |  |
| Women | 701 | 239 | 17.6 | 15.2 | 17.5 | 6.5 | 0.3 |
| Men | 3272 | 1337 | 82.4 | 84.8 | 82.5 | -6.5 | -0.3 |
|  |  |  |  |  |  |  |  |
| **Indigenous status** |  |  |  |  |  |  |  |
| Indigenous | 1087 | 451 | 27.4 | 28.6 | 27.4 | -2.7 | 0.0 |
| Non-Indigenous | 2886 | 1125 | 72.6 | 71.4 | 72.6 | 2.7 | 0.0 |
|  |  |  |  |  |  |  |  |
| **Discharge year** |  |  |  |  |  |  |  |
| 2008 | 384 | 75 | 9.7 | 4.8 | 9.8 | 19.0 | -0.3 |
| 2009 | 494 | 159 | 12.4 | 10.1 | 12.4 | 7.3 | 0.0 |
| 2010 | 452 | 296 | 11.4 | 18.8 | 11.4 | -20.8 | 0.0 |
| 2011 | 357 | 398 | 9 | 25.3 | 9 | -44.3 | 0.0 |
| 2012 | 391 | 197 | 9.8 | 12.5 | 9.5 | -8.6 | 1.0 |
| 2013 | 545 | 118 | 13.7 | 7.5 | 13.5 | 20.2 | 0.6 |
| 2014 | 638 | 144 | 16.1 | 9.1 | 16.1 | 21.2 | 0.0 |
| 2015 | 712 | 189 | 17.9 | 12 | 18.3 | 16.6 | -1.0 |
|  |  |  |  |  |  |  |  |
| **Months in prison** |  |  |  |  |  |  |  |
| 0-6 | 1629 | 777 | 41 | 49.3 | 41 | -16.7 | 0.0 |
| 7-12 | 1059 | 385 | 26.7 | 24.4 | 26.8 | 5.3 | -0.2 |
| 13-18 | 429 | 162 | 10.8 | 10.3 | 10.1 | 1.6 | 2.3 |
| 19-24 | 197 | 73 | 5 | 4.6 | 5.1 | 1.9 | -0.5 |
| 25-30 | 147 | 30 | 3.7 | 1.9 | 3.1 | 10.9 | 3.3 |
| 31-36 | 99 | 40 | 2.5 | 2.5 | 2.7 | 0.0 | -1.3 |
| >36 | 413 | 109 | 10.4 | 6.9 | 11.2 | 12.5 | -2.6 |
|  |  |  |  |  |  |  |  |
| **Prison remoteness** |  |  |  |  |  |  |  |
| Major city | 573 | 1594 | 40.1 | 36.4 | 40.2 | 7.6 | -0.2 |
| Inner regional | 610 | 1617 | 40.7 | 38.7 | 42 | 4.1 | -2.6 |
| Outer regional | 360 | 701 | 17.6 | 22.8 | 16.4 | -13.0 | 3.2 |
| Remote/very remote | 33 | 61 | 1.5 | 2.1 | 1.4 | -4.5 | 0.8 |
|  |  |  |  |  |  |  |  |
| **History of Connections involvement** |  |  |  |  |  |  |  |
| First eligible episode with no previous custody episodes | 470 | 170 | 11.8 | 10.8 | 12.3 | 3.2 | -1.5 |
| First eligible episode with previous custody episodes | 2090 | 886 | 52.6 | 56.2 | 53.9 | -7.2 | -2.6 |
| Subsequent eligible episode with no previous involvement with Connections program | 591 | 204 | 14.9 | 12.9 | 14.1 | 5.8 | 2.3 |
| Subsequent eligible episode with previous involvement with Connections program | 822 | 316 | 20.7 | 20.1 | 19.7 | 1.5 | 2.5 |
|  |  |  |  |  |  |  |  |
| **OAT during current imprisonment episode** |  |  |  |  |  |  |  |
| Yes | 2833 | 1055 | 71.3 | 66.9 | 70.3 | 9.5 | 2.2 |
| No | 1140 | 521 | 28.7 | 33.1 | 29.7 | -9.5 | -2.2 |

**Supplementary Table 2. Probabilities of allocation to Connections versus treatment-as-usual, and distribution of propensity score weights for releasees allocated to treatment-as-usual**

|  |  | **Percentile** | | | **Mean** | **Mini-mum** | **Maxi-mum** |
| --- | --- | --- | --- | --- | --- | --- | --- |
|  |  | **25^th^** | **50^th^** | **75^th^** |  |  |  |
| Probability releasee allocated to Connections | | 0.68 | 0.78 | 0.83 | 0.74 | 0.28 | 0.95 |
| Probability releasee allocated to treatment-as-usual | | 0.53 | 0.66 | 0.78 | 0.65 | 0.26 | 0.92 |
| Weight* applied to releasees allocated to treatment-as-usual | | 1.12 | 1.97 | 3.55 | 2.52 | 0.35 | 12.1 |

*Weights do not preserve the sample size as they, on average, uplift the treatment-as-usual group to match the Connections group. Our use of complex survey weighting ensures that standard errors are not affected by the scale of the weights.

**Supplementary Table 3. Return-to-custody and all-cause mortality within 28 days and within two years of release, by Indigenous status**

|  | **Number** | **Unweighted %** | **Weighted %** | ***Hazard Ratio (95% CI)** |
| --- | --- | --- | --- | --- |
| **Indigenous people** |  |  |  |  |
| **(a) Returned to custody…** |  |  |  |  |
| *Within 28 days of release* |  |  |  |  |
| On Connections | 56 | 5.2 | 5.2 | 2.3 (1.14, 4.65) |
| Treatment-as-usual | 16 | 3.5 | 3.5 | 1 |
|  |  |  |  |  |
| *Within 2 years of release* |  |  |  |  |
| On Connections | 693 | 63.8 | 63.8 | 1.14 (0.97, 1.35) |
| Treatment-as-usual | 273 | 60.5 | 58.2 | 1 |
|  |  |  |  |  |
| **(b) Died…** |  |  |  |  |
| *Within 28 days of release* |  |  |  |  |
| On Connections | 4 | 0.37 | 0.37 | 0.34 (0.07, 1.60) |
| Treatment-as-usual | 5 | 1.11 | 1.07 | 1 |
|  |  |  |  |  |
| *Within 2 years of release* |  |  |  |  |
| On Connections | 31 | 2.85 | 2.85 | 1.18 (0.57, 2.48) |
| Treatment-as-usual | 16 | 3.55 | 2.41 | 1 |
|  |  |  |  |  |
| **Non-Indigenous people** |  |  |  |  |
| **(a) Returned to custody…** |  |  |  |  |
| *Within 28 days of release* |  |  |  |  |
| On Connections | 106 | 3.7 | 3.7 | 0.7 (0.52, 1.12) |
| Treatment-as-usual | 53 | 4.7 | 5.0 | 1 |
|  |  |  |  |  |
| *Within 2 years of release* |  |  |  |  |
| On Connections | 1,196 | 41.4 | 41.4 | 0.96 (0.85, 1.09) |
| Treatment-as-usual | 471 | 41.9 | 42.6 | 1 |
| **(b) Died…** |  |  |  |  |
| *Within 28 days of release* |  |  |  |  |
| On Connections | 6 | 0.21 | 0.21 | 0.40 (0.11, 1.51) |
| Treatment-as-usual | 5 | 0.44 | 0.52 | 1 |
|  |  |  |  |  |
| *Within 2 years of release* |  |  |  |  |
| On Connections | 106 | 3.67 | 3.67 | 0.94 (0.61, 1.44) |
| Treatment-as-usual | 44 | 3.91 | 3.90 | 1 |

**Supplementary Table 4. Started or continued OAT in the community within 28 days of release from prison, by Indigenous status**

|  | **Number** | **Unweighted %** | **Weighted %** | **Odds Ratio** (95% CI) |
| --- | --- | --- | --- | --- |
| **Indigenous** |  |  |  |  |
| On Connections | 628 | 57.8 | 57.8 | 1.10 (0.85 - 1.41) |
| Treatment-as-usual | 228 | 50.6 | 55.5 | 1 |
|  |  |  |  |  |
| **Non-Indigenous** |  |  |  |  |
| On Connections | 1,826 | 63.3 | 63.3 | 1.26 (1.07 - 1.48) |
| Treatment-as-usual | 623 | 55.4 | 57.8 | 1 |

*Excludes people who returned to prison or died within 28 days of release

**Supplementary Table 5. Return-to-custody and all-cause mortality within 28 days and within two years of release, by sex**

|  | **Number** | **Unweighted %** | **Weighted %** | **Hazard Ratio (95% CI)** |
| --- | --- | --- | --- | --- |
| **Women** |  |  |  |  |
| **(a) Returned to custody…** |  |  |  |  |
| *Within 28 days of release* |  |  |  |  |
| On Connections | 28 | 4.0 | 4.0 | 10.87 (2.57 – 46.05) |
| Treatment-as-usual | 2 | 0.8 | 0.4 | 1 |
|  |  |  |  |  |
| *Within 2 years of release* |  |  |  |  |
| On Connections | 249 | 35.5 | 35.5 | 1.29 (0.95 - 1.76) |
| Treatment-as-usual | 76 | 31.8 | 28.1 | 1 |
|  |  |  |  |  |
| **(b) Died…** |  |  |  |  |
| *Within 28 days of release* |  |  |  |  |
| On Connections | 2 | 0.29 | 0.29 | 0.78 (0.07 - 8.68) |
| Treatment-as-usual | 1 | 0.42 | 0.36 | 1 |
|  |  |  |  |  |
| *Within 2 years of release* |  |  |  |  |
| On Connections | 23 | 3.28 | 3.28 | 1.05 (0.39 - 2.81) |
| Treatment-as-usual | 9 | 3.77 | 3.14 | 1 |
| **Men** |  |  |  |  |
| **(a) Returned to custody…** |  |  |  |  |
| *Within 28 days of release* |  |  |  |  |
| On Connections | 134 | 4.1 | 4.1 | 0.83 (0.59, 1.17) |
| Treatment-as-usual | 67 | 5.0 | 5.5 | 1 |
|  |  |  |  |  |
| *Within 2 years of release* |  |  |  |  |
| On Connections | 1,640 | 50.1 | 50.1 | 0.98 (0.88, 1.09) |
| Treatment-as-usual | 668 | 50.0 | 50.9 | 1 |
|  |  |  |  |  |
| **(b) Died…** |  |  |  |  |
| *Within 28 days of release* |  |  |  |  |
| On Connections | 8 | 0.24 | 0.24 | 0.33 (0.11, 0.98) |
| Treatment-as-usual | 9 | 0.67 | 0.74 | 1 |
|  |  |  |  |  |
| *Within 2 years of release* |  |  |  |  |
| On Connections | 114 | 3.48 | 3.48 | 0.98 (0.65, 1.46) |
| Treatment-as-usual | 51 | 3.82 | 3.56 | 1 |

**Supplementary Table 6. Started or continued OAT in the community within 28 days of release from prison, by sex**

|  | **Number** | **Unweighted %** | **Weighted %** | **Odds Ratio (95% CI)** |
| --- | --- | --- | --- | --- |
| **Women** |  |  |  |  |
| On Connections | 454 | 64.8 | 64.8 | 0.89 (0.63 - 1.27) |
| Treatment-as-usual | 155 | 64.9 | 67.3 | 1 |
|  |  |  |  |  |
| **Men** |  |  |  |  |
| On Connections | 2,000 | 61.1 | 61.1 | 1.27 (1.10 - 1.48) |
| Treatment-as-usual | 696 | 52.1 | 55.2 | 1 |

*Excludes people who returned to custody or died within 28 days of release

**Supplementary Table 7. Sensitivity analysis excluding people who were allocated to Connections but who declined to participate: Return-to-custody and all-cause mortality within 2 years of release**

|  | **Number** | **Unweighted %** | **Weighted %** | **Hazard Ratio (95% CI)** |
| --- | --- | --- | --- | --- |
| **(a) Returned to custody…** | |  |  |  |
| *Within 28 days of release* | |  |  |  |
| On Connections* | 121 | 3.8 | 3.8 | 0.93 (0.66-1.31) |
| Treatment as usual | 69 | 4.4 | 4.6 | 1 |
|  |  |  |  |  |
| *Within 2 years of release* | |  |  |  |
| On Connections* | 1511 | 48.0 | 48.0 | 1.03 (0.93-1.14) |
| Treatment as usual | 744 | 47.2 | 47.0 | 1 |
|  |  |  |  |  |
| **(b) Died…** |  |  |  |  |
| *Within 28 days of release* | |  |  |  |
| On Connections* | 10 | 0.32 | 0.32 | 0.48 (0.18-1.30) |
| Treatment as usual | 10 | 0.63 | 0.66 | 1 |
|  |  |  |  |  |
| *Within 2 years of release* | |  |  |  |
| On Connections* | 115 | 3.65 | 3.65 | 1.05 (0.72-1.53) |
| Treatment as usual | 60 | 3.81 | 3.48 |  |

* Excludes people who declined to participate
